# Proton pump inhibitor therapy after transcatheter angiography in refractory nonvariceal acute upper gastrointestinal bleeding patients: a cohort study

**DOI:** 10.1186/s12876-024-03261-4

**Published:** 2024-05-17

**Authors:** Xue Xiao, Xinbing Liu, Hailin Yan, Xiaocun Xing, Xuefeng Luo, Jinlin Yang

**Affiliations:** 1 Department of Gastroenterology and Hepatology, West China Hospital of Sichuan University, 37 GuoXue Xiang, Chengdu, 610041 Sichuan Province China; 2 Sichuan University-University of Oxford Huaxi Joint Centre for Gastrointestinal Cancer, Chengdu, Sichuan Province China; 3 Endoscopy Center, Sichuan Cancer Hospital, Chengdu, Sichuan Province China

**Keywords:** Nonvariceal upper gastrointestinal bleeding, Proton pump inhibitors, Transcatheter angiography

## Abstract

**Background:**

Transcatheter angiography (TA) could help to diagnose and treat refractory nonvariceal upper gastrointestinal bleeding (NVUGIB). Proton pump inhibitors (PPIs) are the key medication for reducing the rebleeding rate and mortality and are usually continued after TA. It is unknown whether high-dose PPIs after TA are more effective than the standard regimen.

**Methods:**

We retrospectively collected data from patients who received TA because of refractory NVUGIB from 2010 to 2020 at West China Hospital. 244 patients were included and divided into two groups based on the first 3 days of PPIs treatment. All baseline characteristics were balanced using the inverse probability of treatment weighting method. The 30-day all-cause mortality, rebleeding rate and other outcomes were compared. The propensity score matching method was also used to verify the results.

**Results:**

There were 86 patients in the high-dose group and 158 in the standard group. The average daily doses of PPI were 192.1 ± 17.9 mg and 77.8 ± 32.0 mg, respectively. Cox regression analysis showed no difference in the 30-day all-cause mortality (aHR 1.464, 95% CI 0.829 to 2.584) or rebleeding rate (aHR 1.020, 95% CI 0.693 to 1.501). There were no differences found in red blood cell transfusion, hospital stay length and further interventions, including endoscopy, repeating TA, surgery and ICU admission. The results were consistent in the subgroup analysis of patients with transcatheter arterial embolization.

**Conclusion:**

In refractory NVUGIB patients who received TA, regardless of whether embolization was performed, high-dose PPI treatment did not provide additional benefits compared with the standard regimen.

**Supplementary Information:**

The online version contains supplementary material available at 10.1186/s12876-024-03261-4.

## Background

Acute nonvariceal upper gastrointestinal bleeding (NVUGIB) is a common clinical emergency. Proton pump inhibitors (PPIs) are the main medicinal treatment. The latest guidelines by the European Society of Gastrointestinal Endoscopy (ESGE) and American Gastroenterological Association (AGA) suggested that patients with suspected NVUGIB should be initially treated with high-dose PPIs [1, 2]. Blood clot formation and stabilization are pH dependent. When given intravenously and at a high dose, PPIs could rapidly render the stomach pH neutral, which is critical for platelet aggregation [3, 4]. High-dose PPI treatment before endoscopy could reduce the need for endoscopic therapy and further reduce the risk of rebleeding after successful endoscopic haemostasis [5–9]. However, some patients are unable to undergo endoscopy due to unstable haemodynamics, and in those who received endoscopic haemostasis, 5-20% of patients experienced rebleeding [9–11].

Transcatheter angiography (TA) is a diagnostic tool and therapeutic method for refractory NVUGIB patients, particularly in those who are haemodynamically unstable or have failed endoscopic haemostasis [12]. In clinical practice, PPI therapy is often continued after TA, but there are no recommendations about PPI regimens. It is unknown whether high-dose PPI could reduce mortality and rebleeding rates compared with standard therapy. In this retrospective study, we collected data from patients who received TA for refractory NVUGIB and used propensity score methods to evaluate the benefits of high-dose PPIs over standard-dose treatment.

## Methods

### Study Design

This was a retrospective cohort study conducted at West China Hospital, Sichuan University. Between May 1, 2010, and April 30, 2020, we identified 288 patients who underwent transcatheter angiography (TA) due to refractory gastrointestinal bleeding, which means haemodynamically unstable after medical treatment or unsuccessful endoscopic haemostasis. We excluded the following patients: (1) 15 due to confirmed lower gastrointestinal or abdominal bleeding; (2) 8 due to oesophagogastric variceal bleeding; (3) 6 due to bleeding from gastrointestinal tumours; (4) 2 due to complications with pathological coagulation disorders; (5) 6 due to hospitalization days exceeding 6 months; and (6) 7 due to incomplete medical data. Ultimately, 244 patients diagnosed with acute nonvariceal upper gastrointestinal bleeding (NVUGIB) were included in the analysis. This study was approved by the Medical Ethics Committee of West China Hospital, Sichuan University, and informed consent was remitted.

**Fig. 1:**
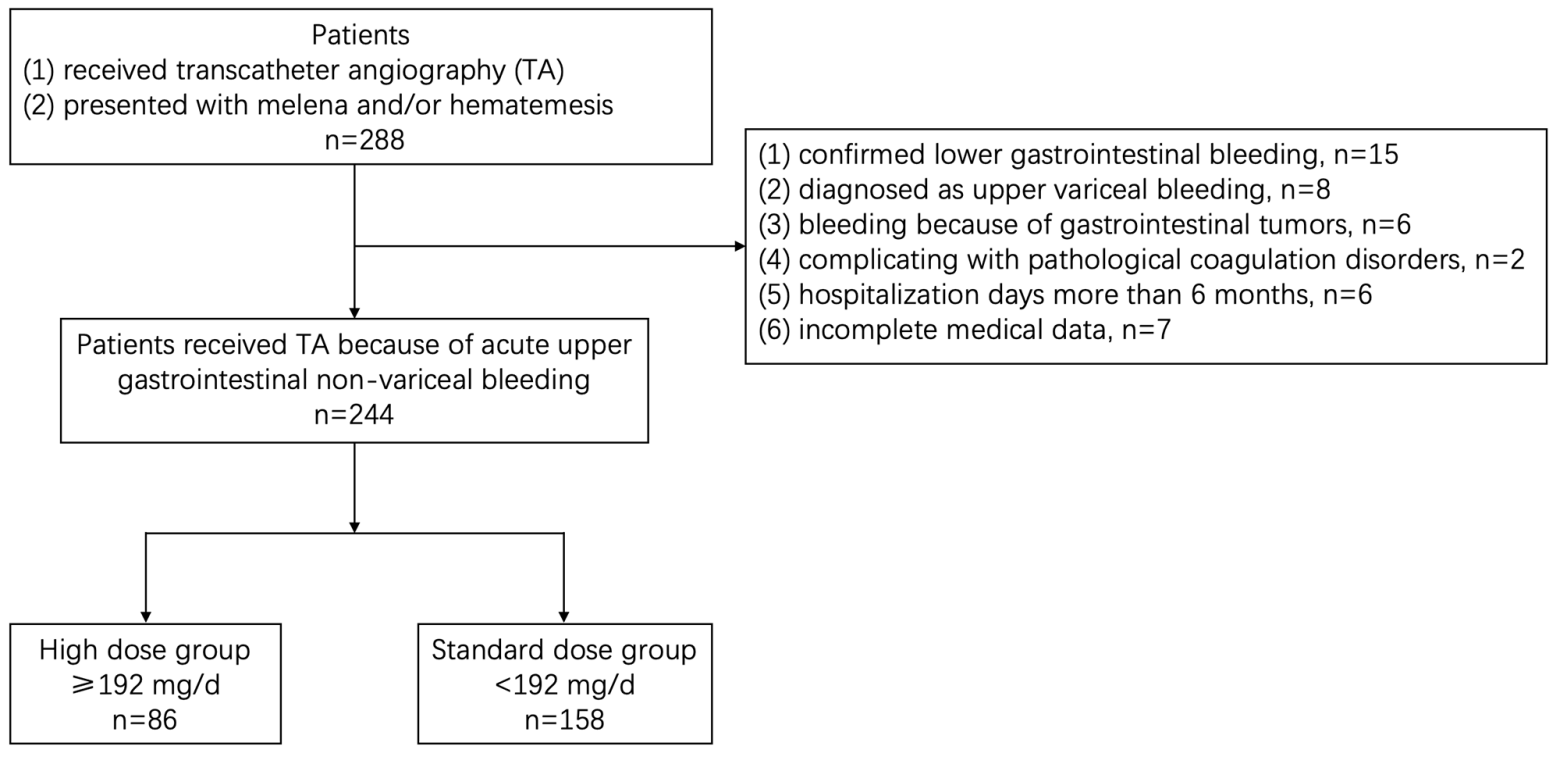
Patients inclusion flowchart

All patients were classified into two groups according to their PPI regimen in the first three days after TA (1). The standard dose group received esomeprazole or omeprazole with a dosage of < 192 mg/d (usually 40 mg infusion once or twice per day); (2) The high dose group received esomeprazole or omeprazole with a dosage of ≥ 192 mg/d (usually 8 mg/h continuous infusion). The study flowchart is described in Fig. 1.

### Data collection

The baseline was taken at the time of TA. Bleeding severity was retrospectively assessed with the Glasgow-Blatchford score (GBS) and Shock index (SI). The PPI regimen and medical history, including peptic ulcer history, gastrointestinal bleeding history, comorbidities, medicine taken in the recent 1 month before bleeding, whether endoscopy or computed tomography angiography (CTA) examination was performed before TA, were collected from the database of the Hospital Information System (HIS).

The 30-day all-cause mortality was collected as the primary endpoint. If the patient was discharged early before the set date, our investigators would call the patient or his or her kin for more information. Usually, the discharged patients would ask to come back two weeks later for drug prescription, and the outpatient data were also recorded in the HIS. Secondary outcomes included (1) 30-day rebleeding rate; (2) need for surgery within hospitalization; (3) need for therapeutic endoscopy within hospitalization; (4) need for further angiography within hospitalization; (5) need for intensive care unit (ICU) admission within hospitalization; (6) length of hospital stay; and (7) units of blood red cell transfusion within hospitalization.

### Statistical analysis

All statistical data were analysed using SPSS version 23.0 and R Project for Statistical Computing software, V.3.6.0. The baseline characteristics were adjusted with the inverse probability of treatment weighting (IPTW) method. A standardized mean difference (SMD) ≤ 0.1 denotes a good balance of covariates. The balanced characteristics included sex, age, GBS, SI, haemoglobin concentration, ulcer history, bleeding history, medicine history (anticoagulation, nonsteroid anti-inflammatory drugs, steroids), gastrointestinal surgery history, comorbidities of cirrhosis or liver failure, chronic kidney disease, gastrointestinal cancer and cardiac disease, and whether they received endoscopy or CTA examination before TA. The chi-square test was used to analyse differences in proportions. Student’s t test and Mann–Whitney U test were used for parametric and nonparametric data, respectively. Cox proportional hazards regression was applied to analyse the 30-day all-cause mortality and rebleeding rate, and the Schoenfeld residual test verified the assumption of proportional hazards. For the condition of violation of proportional hazards (the 30-day rebleeding rate in the subgroup of targeted embolization during TA), a time-dependent analysis was used. These two endpoints were also analysed with the Kaplan‒Meier method. Variables were selected for multivariate analysis with a p value cut-off ≤ 0.1. However, if there were fewer than 3 variables in the univariate analysis with *p* ≤ 0.1, a p value cut-off ≤ 0.5 was used instead. In sensitivity analyses, the groups were balanced again with propensity score matching (PSM) methods. The propensity score variables included age, sex, comorbidities, conditions of endoscopy and CTA, GBS, SI and haemoglobin concentration. The greedy nearest neighbour algorithm was used, and patients were matched in a 1:1 fashion using a calliper width of 0.2 standard deviations of logit of the estimated propensity score.

## Results

We analysed data from 244 patients who underwent transcatheter angiography (TA) because of refractory nonvariceal upper gastrointestinal bleeding (NVUGIB). There were 158 patients assigned to the standard-dose group and 86 patients to the high-dose group (Table 1). The average daily PPI dose was 192.1 mg (SD = 17.9) in the high-dose group and 77.8 mg (SD = 32.0) in the standard-dose group. In the high-dose group, 82.6% (71/86) of patients received transcatheter arterial embolization, in which 81.7% (58/71) of patients received targeted embolization to the offending vessels, including vessels with active contrast agent extravasation and arterial aneurysm formation. In the standard dose group, 78.5% (124/158) of patients received embolization during TA, and 82.3% (102/124) received targeted embolization (Supplementary Table 1). The gastroduodenal artery, pancreaticoduodenal artery and left gastric artery were the three most commonly embolized arteries in both groups (Supplementary Table 1).

**Table 1:** Patients’ characteristics before and after IPTW

	Unmatch			IPTW		
	High dose *n* = 86	Standard dose *n* = 158	SMD	High dose *n* = 86	Standard dose *n* = 158	SMD
Male (%)	74 (86.0)	120 (75.9)	0.260	68.2 (79.3)	125.7 (79.6)	0.007
Age (mean (SD))	55.99 (15.60)	56.23 (16.18)	0.015	56.76 (14.70)	56.34 (16.31)	0.027
Peptic Ulcer history (%)	17 (19.8)	27 (17.1)	0.069	17.2 (20.0)	29.6 (18.8)	0.032
Gastrointestinal bleeding history (%)	20 (23.3)	32 (20.3)	0.073	19.6 (22.8)	34.6 (21.9)	0.021
Medicine history (%)			0.114			0.033
Anticoagulation	2 (2.3)	2 (1.3)		1.3 (1.5)	2.4 (1.5)	-
NSAID	9 (10.5)	17 (10.8)		10.0 (11.6)	17.4 (11.0)	-
Steroid	2 (2.3)	2 (1.3)		1.5 (1.7)	3.4 (2.1)	-
None	73 (84.9)	137 (86.7)		73.2 (85.1)	134.8 (85.3)	-
Cardiac diseases ^1^(%)	9 (10.5)	13 (8.2)	0.077	9.5 (11.0)	15.6 (9.9)	0.038
Liver diseases ^2^(%)	11 (12.8)	36 (22.8)	0.264	18.4 (21.4)	30.7 (19.4)	0.049
Chronic kidney diseases (%)	14 (16.3)	11 (7.0)	0.294	9.9 (11.5)	17.2 (10.9)	0.018
Gastrointestinal Cancer (%)	15 (17.4)	48 (30.4)	0.307	20.9 (24.3)	40.3 (25.5)	0.027
Gastrointestinal surgery history (%)	24 (27.9)	74 (46.8)	0.399	24 (27.9)	74 (46.8)	0.037
Endoscopy (%)	73 (84.9)	110 (69.6)	0.370	62.3 (72.4)	116.9 (74.0)	0.034
CTA examination (%)	17 (19.8)	27 (17.1)	0.069	17 (19.8)	27 (17.1)	0.023
GBS (mean (SD))	12.10 (2.95)	10.94 (3.00)	0.392	11.46 (2.98)	11.38 (3.08)	0.026
SI (mean (SD))	1.00 (0.39)	0.93 (0.31)	0.198	0.94 (0.35)	0.94 (0.33)	0.003
HB concentration (mean (SD))	64.53 (16.90)	69.51 (20.44)	0.265	67.60 (17.49)	67.83 (19.83)	0.012

^1^ Cardiac diseases include heart failure and ischemic heart disease

^2^ Liver diseases include cirrhosis and liver failure

SD, standard deviation; CTA, computed tomography angiography; IPTW, inverse probability of treatment weighting; SMD, Standardized mean difference; GBS, Glasgow-Blatchford score; SI, Shock index; HB, hemoglobin; NSAID, non-steroid anti-inflammatory drug

Before TA, there was a higher ratio of patients in the high-dose group who received endoscopy than in the standard-dose group (84.9% vs. 69.6%, Table 1). With endoscopy, 87.7% (64/73) of patients in the high-dose group were diagnosed with peptic ulcer-related bleeding, and the ratio in the standard-dose group was 80.9% (89/110). The endoscopic haemostasis ratio was 46.6% (34/73) in the high-dose group and 50% (55/110) in the standard-dose group (Supplementary Table 2). IPTW was used to balance all the baseline data, including medical history, drug history, complications and bleeding severity. After balancing, GBS and SI were situated at scores of approximately 11 and 1, respectively (Table 1).

### The 30-day all-cause mortality

**Fig. 2:**
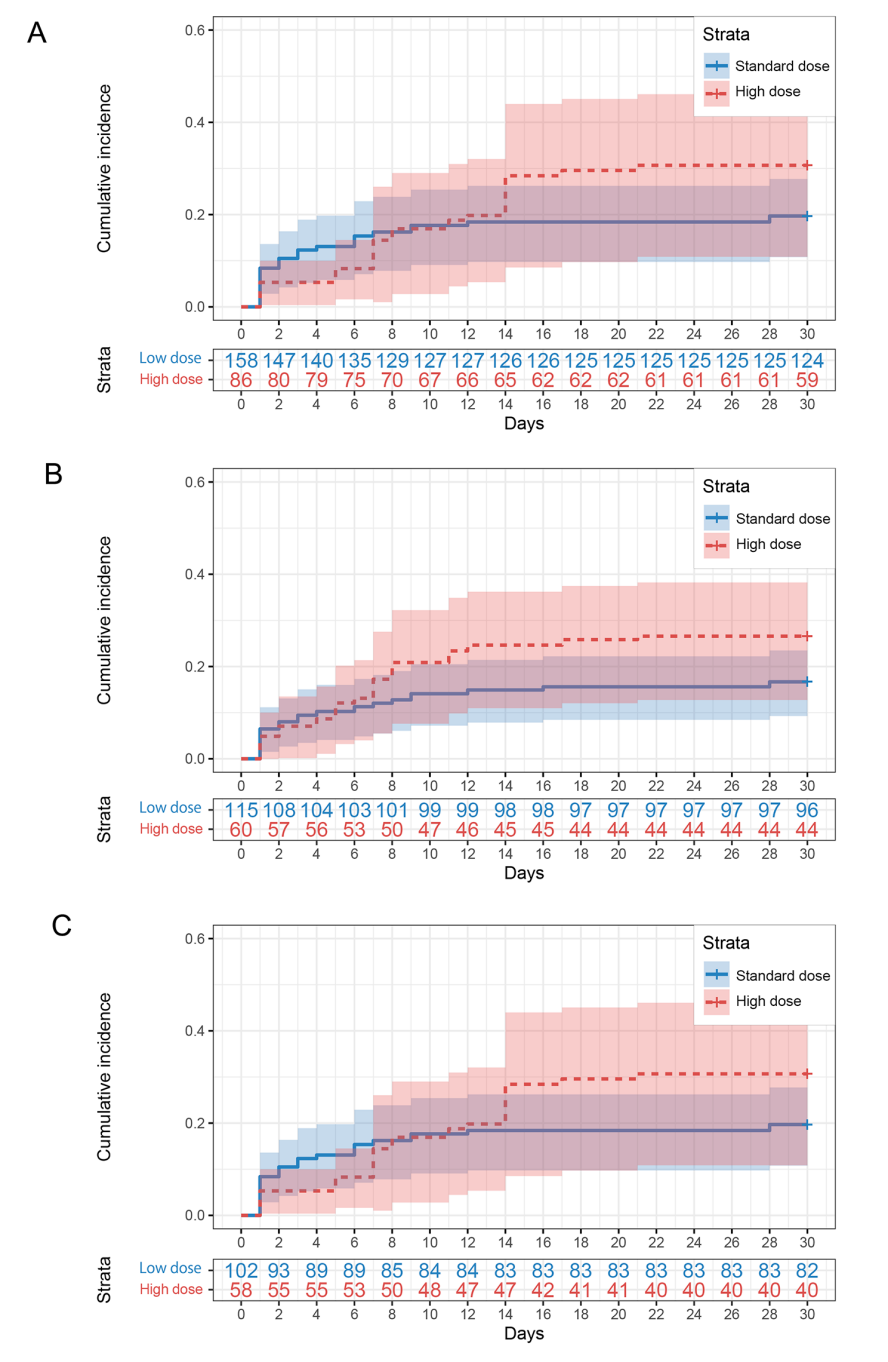
Kaplan-Meier plots for 30-day all-cause mortality in (**A**) patients received TA, (**B**) patients received embolization during TA, (**C**) patients received targeted embolization to offending vessels. The number chart below each plot was the corresponding survival patient number. TA, transcatheter angiography

In the Cox regression analysis, we found no difference in the 30-day all-cause mortality between the high-dose and standard-dose groups (aHR 1.464, 95% CI 0.829 to 2.584, *p* = 0.189) (Fig. 2; Table 2). The 30-day all-cause mortality was 27.9% (24/86) and 20.3% (32/158) in the two groups, respectively. In the high-dose group, 58.3% (14/24) of patients died in 7 days, compared with 78.1% (25/32) in the standard-dose group. After balancing the baseline characteristics, there was still no difference in 7-day all-cause mortality between the two groups (*p* = 0.613).

**Table 2:** Outcome of 30-day all-cause mortality

			Univariate		Multivariate	
	No. of patients	No. of events	HR(95%CI)	*P* value	aHR(95%CI)	*P* value
TA						
High dose	86	24	1.340 (0.820,2.570)	0.201	1.464 (0.829,2.584)	0.189^Ú^
Standard dose	158	32	1	reference	1	reference
TAE						
High dose	60	16	1.640 (0.798,3.360)	0.179	1.730 (0.756, 3.959)	0.195^†^
Standard dose	115	19	1	reference	1	reference
Targeted TAE						
High dose	58	15	1.540 (0.700, 3.380)	0.284	1.347 (0.651, 2.789)	0.423^‡^
Standard dose	102	19	1	reference	1	reference

^Ú^: For multivariate analysis, the adjusting factors included age, medicine history, chronic kidney diseases, gastrointestinal cancer, cardiac diseases, endoscopy, GBS and SI.

^†^: For multivariate analysis, the adjusting factors included liver diseases, gastrointestinal cancer, cardiac diseases, endoscopy, GBS and SI.

^‡^: For multivariate analysis, the adjusting factors included gastrointestinal cancer, cardiac diseases, endoscopy, GBS and SI.

TA, Transcatheter angiography; TAE, transcatheter arterial embolization; CI, confidence interval; HR, hazard ratio; aHR, adjusted hazard ratio

### The 30-day rebleeding rates and red blood cell transfusion units

**Fig. 3:**
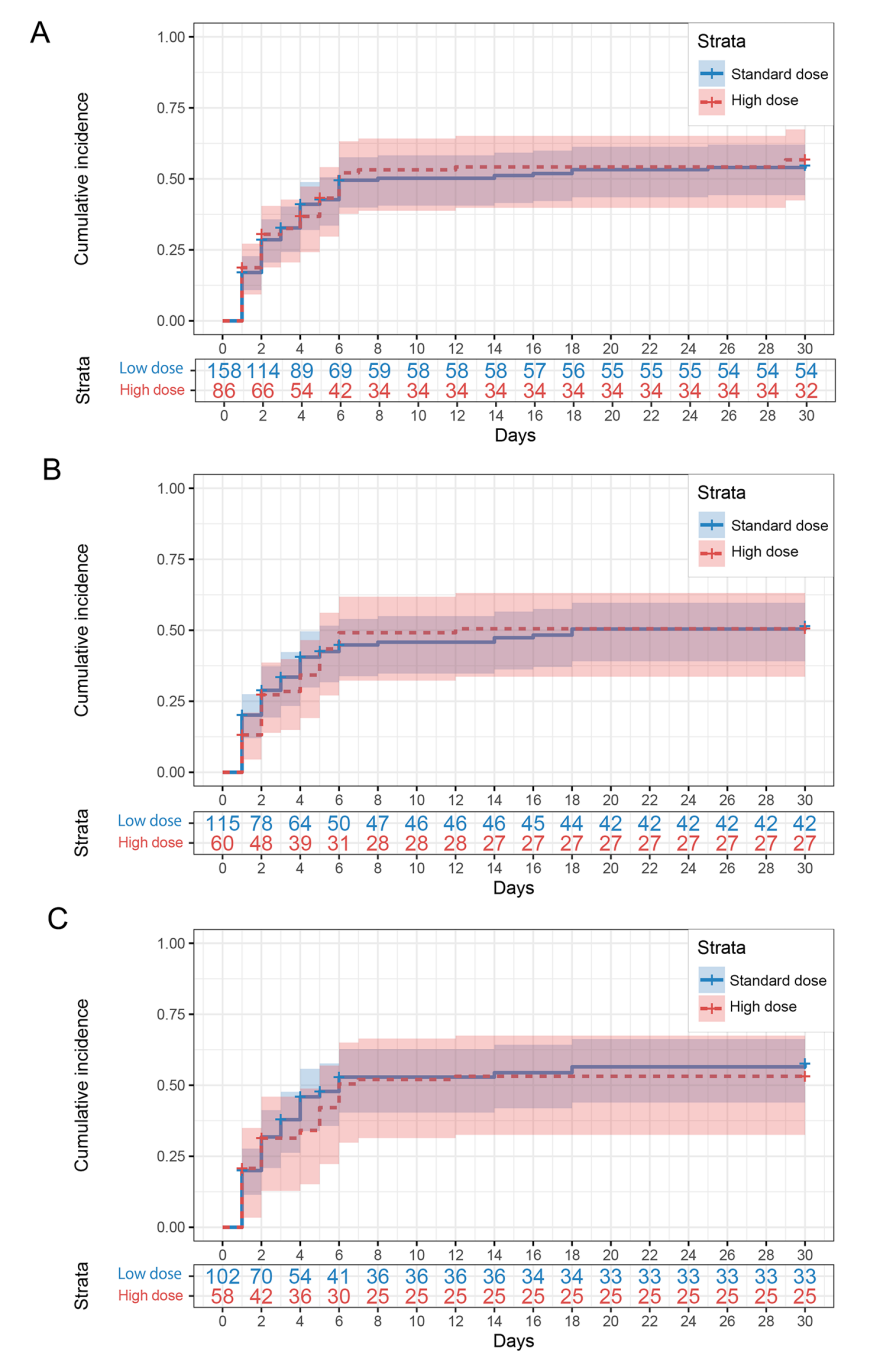
Kaplan-Meier plots for in-hospital rebleeding rate in (**A**) patients received TA, (**B**) patients received embolization during TA, (**C**) patients received targeted embolization to offending vessels. The number chart below each plot was the corresponding patient number without recurrent bleeding after TA. TA, transcatheter angiography

The Cox regression analysis showed no differences in the 30-day rebleeding rate between the two groups (aHR 1.020, 95% CI 0.693 to 1.501, *P* = 0.921) (Fig. 3; Table 3). In all refractory bleeding patients who received TA, 48.0% (117/244) of patients experienced recurrent bleeding during hospitalization. In the high-dose group, the 30-day rebleeding rate was 51.2% (44/86), and 95.5% (42/44) occurred in the first 7 days after TA. In the standard dose group, the 30-day rebleeding rate was 46.2% (73/158), and 93.2% (68/73) occurred in the first 7 days. During hospitalization, the average transfusion units of red blood cells were 5.3 in the high-dose group and 4.3 in the standard-dose group. After balancing the baseline data, no differences between the two groups were found (*p* = 0.406) (Supplementary Table 3).

**Table 3:** Outcome of 30-day rebleeding rate

			Univariate		Multivariate	
	No. of patients	No. of events	HR(95%CI)	*P* value	aHR(95%CI)	*P* value
TA						
High dose	86	44	1.047(0.713,1.537)	0.816	1.020 (0.693,1.501)	0.921^Ú^
Standard dose	158	73	1	reference	1	reference
TAE						
High dose	60	27	0.934 (0.584,1.494)	0.776	0.866 (0.533,1.406)	0.561^†^
Standard dose	115	50	1	reference	1	reference
Targeted TAE						
High dose	58	27	0.843 (0.626, 1.136)	0.262	0.781 (0.557, 1.094)	0.150^‡^
Standard dose	102	48	1	reference	1	reference

^Ú^: For multivariate analysis, the adjusting factors included sex, age, gastrointestinal cancer, CTA examination, GBS and SI.

^†^: For multivariate analysis, the adjusting factors included age, liver diseases, chronic kidney diseases, gastrointestinal cancer, CTA examination, GBS and SI.

^‡^: For multivariate analysis, the adjusting factors included age, chronic kidney diseases, gastrointestinal surgery history, endoscopy, CTA examination, GBS and SI.

TA, Transcatheter angiography; TAE, transcatheter arterial embolization; CI, confidence interval; HR, hazard ratio; aHR, adjusted hazard ratio

### The need for further treatments in surgical operation, therapeutic endoscopy and repeating TA

After TA, the rebleeding patients received further interventions. In the high-dose group, 12.8% (11/86) of patients received therapeutic endoscopy, 7.0% (6/86) received a second TA, and 16.3% (14/86) received surgery. In the standard dose group, 20.88% (33/158) of patients had therapeutic endoscopy, 6.3% (10/158) received repeated TA, and 20.3% (32/158) underwent surgical operation. After balancing the baseline data, there were no differences found between the two groups in the need for further treatments, including therapeutic endoscopy, repeating TA and surgical operation (Supplementary Table 3). However, when focusing on the data at 7 days, we found that 7 (8.1%) patients in the high-dose group received endoscopy haemostasis compared with 29 (18.4%) in the standard-dose group (chi-square test, *p* = 0.029).

### The need for ICU admission within hospitalization and hospital stay length

The average hospital stay was 23.9 days in the high-dose group and 20.7 days in the standard group. A total of 46.5% (40/86) of patients in the high-dose group were admitted to the ICU, compared with 31.65% (50/158) in the standard-dose group. No differences between the two groups were evidenced in the statistical analysis (Supplementary Table 3).

### Subgroup analysis

We analysed subgroups based on patients who received embolization (TAE, including empirical embolization and targeted embolization) and the subgroups of patients with targeted embolization to offending vessels (targeted TAE). These two subgroups’ baseline characteristics were also balanced with IPTW (Supplementary Tables 4 and Supplementary Table 5). We obtained congruent results in which there were no differences between the high-dose and standard-dose groups in the 30-day all-cause mortality and rebleeding rates (Tables 2 and 3; Figs. 2 and 3). In both the TAE and targeted TAE subgroup analyses, the therapeutic endoscopy rate in the first 7 days was lower in the high-dose group than in the standard-dose group (4.0% vs. 18.7%, *p* = 0.005 after IPTW; 5.8% vs. 19.7%, *p* = 0.015 after IPTW). However, significant differences in the 30-days therapeutic endoscopy rate were not found. In patients with targeted TAE, high-dose PPI treatment increased the hospital stay length (25.47 ± 18.05 vs. 21.11 ± 20.00, *p* = 0.015 after IPTW). No significant differences were found in other secondary endpoints in the two subgroup analyses (Supplementary Tables 6–7).

### Sensitivity analyses

To test the reliability of our results, we analysed the cohort with PMS again and observed consistent results that there were no differences in the 30-day all-cause mortality and rebleeding rate between the high-dose and standard-dose PPI groups (Supplemental Table 8). We further restricted the analysis to patients without gastrointestinal cancer, and the final Cox regression results were congruent with the original results (Supplemental Table 9).

## Discussion

Our findings demonstrate that high-dose PPI treatment in acute nonvariceal upper gastrointestinal bleeding patients after TA did not reduce the 30-day all-cause mortality and recurrent bleeding rate compared with standard-dose therapy and did not improve overall outcomes.

The aim of TA is to treat refractory NVUGIB patients who failed endoscopic haemostasis or had unstable haemodynamics. These patients usually have severe haemorrhage conditions, as shown in our study with a high GBS score and shock index. The high-dose PPI regimen in our study is equal to therapy of not less than 8 mg/h omeprazole or esomeprazole per day, which was also recommended in patients with high-risk stigmata of bleeding in the first 3 days after endoscopy [1, 2, 7]. However, high-dose PPI after TA did not improve the outcomes. This could be explained by the poor response of refractory bleeding patients to high-dose PPI treatment in previous therapy, which was also the reason for refractory bleeding and the need for TA. On the other hand, directly blocking the vessel during TA might weaken the need for a neutral gastric pH value. Therefore, in our subgroup analysis of the embolization patients, high-dose PPI also did not show any benefits in the main prognosis over the standard regimen, including mortality, rebleeding and further intervention.

Recurrent bleeding is an independent risk factor for NVUGIB patient survival [13. For patients with recurrent bleeding after endoscopic haemostasis, both TA and surgery are salvage therapies. A meta-analysis including 1077 refractory bleeding patients demonstrated that transcatheter angiographic embolization is as effective as surgery, but there is a significant reduction in complications with embolization [14. For patients with recurrent bleeding after TA, there are no definitely effective therapies to ameliorate the prognosis. The pooled mortality, including 30-day and in-hospital all-cause mortality in transcatheter angiographic embolization patients, was reported to be 21.8% [14, similar to our patients. Our study showed that the majority of deaths after TA occurred in the first 7 days.

The total embolization rate in our cohort was 71.7%, 65.6% was targeted embolization, and the others were empirical embolization. A meta-analysis including 13 studies concluded that empiric embolization was as safe and effective as targeted embolization, and the rebleeding rate within 30 days ranged from 46.9 to 4.5% [15. These studies included patients with prophylactic embolization. Our study aimed to include ongoing or recurrent bleeding patients and had a larger population than the aforementioned studies. The 30-day rebleeding rate was relatively higher (44% in the embolization cohort, 48% in patients received TA when the embolization situation was not considered).

Although statistical analysis showed that a high-dose regimen reduced endoscopic haemostasis within 7 days in patients with embolization, we could not conclude that high-dose PPI treatment is better in these outcomes. It is necessary to note that patients with recurrent bleeding after TA receive repeated TA or surgery other than endoscopy, and deteriorated patients usually have no chance for endoscopy.

Complicated gastrointestinal cancer increases the risk of death in NVUGIB patients [16, 17]. We excluded cancer patients in the sensitivity analysis, and high-dose PPI treatment also did not show any advantages in the final prognosis compared with the standard-dose group. In addition, the PSM cohort yielded congruent results, which suggests that the final conclusion was reliable.

There is growing evidence to support the use of nonhigh-dose PPIs in NVUGIB. A meta-analysis including 7 randomized controlled studies found that high-dose PPIs (continuous infusion doses > 192 mg/d) did not reduce rebleeding, surgical intervention or mortality in patients after endoscopy [18. The Cochrane review of 22 randomized trials showed no superiority of high-dose PPI treatment (≥ 600 mg/72 h) over a standard dose in peptic ulcer bleeding [19. High-dose PPI therapy as a continuous infusion would not increase the mean intragastric pH and duration of pH > 6, compared with the regimen of intermittent administration every 12 h [20. Unlike the spring-up studies about PPI strategy after endoscopy, there is little data about how to manage PPI after TA in NVUGIB. This study included refractory bleeding patients and revealed that there was no superiority of high-dose PPI therapy over standard-dose treatment after TA, regardless of whether embolization was performed.

We acknowledge weaknesses in this study. The cohort did not include patients with prophylactic embolization who had successful endoscopic haemostasis, and high-dose PPI treatment might be beneficial. The generalizability of our study was limited in these patients. Second, some patients who died early after TA had no chance of finishing endoscopy, and the aetiology diagnosis was not accessible for everyone. The aetiology might be a potential confounder. Moreover, the data were collected retrospectively, recall bias could not be avoided and there might be unidentified or uncontrolled confounders. This study collected data over 10 years, for which the technology advantage might bring a higher embolization rate (including empirical embolization). Nonetheless, we did not think that the embolization rate would change the final conclusion, as has been demonstrated in the subgroup of embolization patients. In the data analysis, we computed the independent variables with IPTW to balance the basic characteristics, which might reduce the influence of confounders and allowed a better demonstration.

## Conclusion

In this study, we found that in refractory NVUGIB patients, including those with recurrent bleeding and initial failed haemostasis, high-dose PPI treatment after TA was not superior to the standard-dose regimen in reducing the 30-day all-cause mortality and rebleeding rate, regardless of embolization.

### Electronic supplementary material

Below is the link to the electronic supplementary material.

Supplementary Material 1

## Data Availability

The datasets used during the current study are available from the corresponding author on reasonable request.

## Abbreviations

TA: Transcatheter angiography
NVUGIB: nonvariceal upper gastrointestinal bleeding
PPIs: Proton pump inhibitors
GBS: Glasgow-Blatchford score
SI: Shock index
CTA: computed tomography angiography
IPTW: Inverse probability of treatment weighting
SMD: Standardized mean difference
PSM: Propensity score matching
TAE: transcatheter arterial embolization
