# Supplementary material for: Proton pump inhibitor therapy after transcatheter angiography in refractory nonvariceal acute upper gastrointestinal bleeding patients: a cohort study
Source: BMC Gastroenterol. 2024 May 17;24:168. doi: 10.1186/s12876-024-03261-4 (PMC11100103; doi:10.1186/s12876-024-03261-4)
Supplement: Supplementary file 1 — Supplementary Material 1 [file 12876_2024_3261_MOESM1_ESM.docx]

**Supplementary Table 1: Transcatheter angiography results**

|  | High dose  n=86 | Standard dose  n=158 |
| --- | --- | --- |
| Transcatheter angiography findings |  |  |
| Active bleeding or Pseudoaneurysm (%) | 58 (67.4) | 102 (64.6) |
| No lesion identified (%) | 28 (32.6) | 56 (35.4) |
| Transcatheter arterial embolization (%) | 71 (82.6) | 124 (78.5) |
| Targeted embolization (%) | 58 (81.7) | 102 (82.3) |
| Gastroduodenal artery | 23 | 38 |
| Pancreaticoduodenal artery | 7 | 22 |
| Left gastric artery | 9 | 14 |
| Right gastric artery | 1 | 4 |
| Arteriae gastricae breves | 0 | 2 |
| Mesenteric artery | 3 | 3 |
| Left hepatic artery | 1 | 1 |
| Right hepatic artery | 1 | 3 |
| Hepatic artery | 6 | 9 |
| Gastroepiplotic artery | 3 | 1 |
| Phrenicartery | 1 | 0 |
| Spleen artery | 3 | 5 |
| Experienced embolization (%) | 13 (18.3) | 22 (17.7) |
| Gastroduodenal artery | 7 | 17 |
| Pancreaticoduodenal artery | 1 | 1 |
| Left gastric artery | 1 | 2 |
| Right gastric artery | 0 | 1 |
| Hepatic artery | 2 | 1 |
| Gastroepiplotic artery | 2 | 0 |

**Supplementary Table 2: Endoscopy results before transcatheter angiography**

|  | High dose  n=86 | Standard dose  n=158 |
| --- | --- | --- |
| Endoscopy before transcatheter angiography (%) | 73 (84.9) | 110 (69.6) |
| Gastric ulcer | 19 | 20 |
| Duodenal ulcer | 31 | 36 |
| Anastomotic ulcer | 4 | 14 |
| Multiple peptic ulcer | 10 | 19 |
| Mallory-Weiss syndrome | 2 | 2 |
| Duodenal diverticulum | 1 | 1 |
| Papilla bleeding | 1 | 5 |
| Endoscopic submucosal dissection | 1 | 0 |
| No lesion identified | 4 | 13 |
| Endoscopic hemostasis (%) | 34 (46.6) | 55 (50.0) |

**Supplementary table 3: Outcome of other secondary endpoints**

|  | High dose  n = 86 | Standard dose  n = 158 | p value | p* value |
| --- | --- | --- | --- | --- |
| Red blood cell transfusion units within 30 days(mean (SD)) | 5.26 (6.09) | 4.29 (4.75) | 0.547 | 0.406 |
| Length of hospital stay (mean (SD)) | 23.94 (19.6) | 20.68 (18.11) | 0.233 | 0.124 |
| Surgery operation within 30 days (%) | 14 (16.3) | 32 (20.3) | 0.497 | 1.000 |
| Therapeutic endoscopy within 30 days (%) | 11 (12.8) | 33 (20.9) | 0.163 | 0.154 |
| Repeating TA within 30 days (%) | 6 (7.0) | 10 (6.3) | 1.000 | 0.949 |
| ICU admission (%) | 40 (46.5) | 50 (31.7) | 0.516 | 0.235 |

p value was calculated before IPTW; p* value was calculated after IPTW.

SD, standard deviation; ICU, intensive care unit; TA, transcatheter angiography.

**Supplementary table 4: Patients’ characteristics before and after IPTW for subgroup of TAE**

|  | **Unmatch** | | | **IPTW** | | |
| --- | --- | --- | --- | --- | --- | --- |
|  | High dose  n = 60 | Standard dose  n = 115 | SMD | High dose  n = 60 | Standard dose  n= 115 | SMD |
| Male (%) | 53 (88.3) | 89 (77.4) | 0.293 | 50.4（84.0） | 93.5（81.3） | 0.07 |
| Age (mean (SD)) | 56.98 (16.09) | 55.61 (15.54) | 0.087 | 55.80（16.17） | 55.91（15.5） | 0.007 |
| Peptic Ulcer history (%) | 13 (21.7) | 23 (20.0) | 0.041 | 14.9（24.8） | 25.1（21.8） | 0.07 |
| Gastrointestinal bleeding history (%) | 16 (26.7) | 27 (23.5) | 0.074 | 17.0（28.3） | 29.6（25.7） | 0.057 |
| Medicine history (%) |  |  | 0.190 |  |  | 0.986 |
| Anticoagulation | 2 (3.3) | 1 (09) |  | 1.0 (1.7) | 1.7 (1.5) | - |
| NSAID | 6 (10.0) | 11 (9.6) |  | 7.2（12.0） | 11.8（10.3） | - |
| Steroid | 1 (1.7) | 1 (0.9) |  | 0.6（1.0） | 1.2 (1.0) | - |
| None | 51(85.0) | 102 (88.7) |  | 51.2（85.3） | 100.3（87.2） | - |
| Cardiac diseases ^1^(%) | 6 (10.0) | 10 (8.7) |  | 5.8（9.7） | 10.4（9.0） | 0.024 |
| Liver diseases ^2^(%) | 9 (15.0) | 21 (18.3) |  | 10.9（18.1） | 20.2（17.6） | 0.941 |
| Chronic kidney diseases (%) | 10 (16.7) | 8 (7.0) |  | 6.6（11.0） | 10.7（9.3） | 0.057 |
| Gastrointestinal Cancer (%) | 10 (16.7) | 33 (28.7) |  | 13.1（21.9） | 28（24.3） | 0.058 |
| Gastrointestinal surgery history (%) | 16 (26.7) | 51 (44.3) |  | 21.2（35.3） | 43.7（38.0） | 0.056 |
| Endoscopy (%) | 50 (83.3) | 83 (72.2) |  | 46.1 (76.9) | 86.6 (75.3) | 0.038 |
| CTA examination (%) | 10 (16.7) | 16 (13.9) |  | 7.5 (12.5) | 16.7 (14.5) | 0.057 |
| GBS (mean (SD)) | 12.3 (3.12) | 11.03 (3.06) |  | 11.69 (3.14) | 11.43 (3.06) | 0.083 |
| SI (mean (SD)) | 1.04 (0.43) | 0.95 (0.32) |  | 0.97 (0.40) | 0.94 (0.34) | 0.009 |
| HB concentration (mean (SD)) | 62.38 (17.55) | 69.46 (21.80) |  | 66.1 (18.93) | 67.23 (21.43) | 0.058 |

^1^: Cardiac diseases include heart failure and ischemic heart disease.

^2^: Liver diseases include cirrhosis and liver failure.

SD, standard deviation; CTA, computed tomography angiography；IPTW, inverse probability of treatment weighting; SMD, Standardized mean difference; GBS, Glasgow-Blatchford score; SI, Shock index; HB, hemoglobin; NSAID, non-steroid anti-inflammatory drug.

**Supplementary table 5: Patients’ characteristics before and after IPTW for subgroup of targeted TAE**

|  | **Unmatch** | | | **IPTW** | | |
| --- | --- | --- | --- | --- | --- | --- |
|  | High dose  n = 58 | Standard dose  n = 102 | SMD | High dose  n = 58 | Standard dose  n= 102 | SMD |
| Male (%) | 54（93.1） | 77（75.5） | 0.499 | 47 (81.0) | 83.3 (81.7) | 0.018 |
| Age (mean (SD)) | 55.67（16.68） | 55.81（15.19） | 0.009 | 57.60 (16.45) | 55.81 (15.17) | 0.113 |
| Peptic Ulcer history (%) | 13（22.4） | 14（13.7） | 0.227 | 9.8 (16.9) | 17.6 (17.2) | 0.008 |
| Gastrointestinal bleeding history (%) | 14（24.1） | 18（17.6） | 0.160 | 11 (18.9) | 20.8 (20.4) | 0.036 |
| Medicine history (%) | - | - | 0.095 | - | - | 0.026 |
| Anticoagulation | 1 (1.7) | 1 (1.0) | - | 0.6 (1.1) | 1.2 (1.2) | - |
| NSAID | 6 (10.3) | 10 (9.8) | - | 6.4 (11.1) | 10.5 (10.3) | - |
| Steroid | 1 (1.7) | 1 (1.0) | - | 0.4 (0.8) | 0.8 (0.8) | - |
| None | 50 (86.2) | 90 (88.2) | - | 50.5 (87.0) | 89.5 (87.7) | - |
| Cardiac diseases ^1^(%) | 5 (8.6) | 8 (7.8) | 0.028 | 8.3 (14.3) | 8.8 (8.7) | 0.178 |
| Liver diseases ^2^(%) | 5 (8.6) | 22 (21.6) | 0.368 | 9.1 (15.7) | 16.9 (16.6) | 0.023 |
| Chronic kidney diseases (%) | 8 (13.8) | 7 (6.9) | 0.229 | 5.7 (9.8) | 8.7 (8.6) | 0.044 |
| Gastrointestinal Cancer (%) | 10 (17.2) | 31 (30.4) | 0.312 | 15.4 (26.5) | 25.5 (25.0) | 0.035 |
| Gastrointestinal surgery history (%) | 12 (20.7) | 48 (47.1) | 0.580 | 23 (39.7) | 38.3 (37.5) | 0.044 |
| Endoscopy (%) | 50 (86.2) | 72 (70.6) | 0.387 | 39.3 (67.8) | 76.2 (74.7) | 0.153 |
| CTA examination (%) | 10 (17.2) | 13 (12.7) | 0.126 | 6.4 (11.1) | 15.1 (14.8) | 0.109 |
| GBS (mean (SD)) | 11.86 (3.18) | 11.14 (3.00) | 0.235 | 11.6 (3.12) | 11.42 (3.00) | 0.059 |
| SI (mean (SD)) | 1.04 (0.42) | 0.96 (0.32) | 0.208 | 0.97 (0.38) | 0.98 (0.34) | 0.042 |
| HB concentration (mean (SD)) | 63.97 (18.99) | 69.16 (20.224) | 0.265 | 68.27 (19.85) | 67.38 (19.71) | 0.045 |

^1^: Cardiac diseases include heart failure and ischemic heart disease.

^2^: Liver diseases include cirrhosis and liver failure.

SD, standard deviation; CTA, computed tomography angiography；IPTW, inverse probability of treatment weighting; SMD, Standardized mean difference; GBS, Glasgow-Blatchford score; SI, Shock index; HB, hemoglobin; NSAID, non-steroid anti-inflammatory drug.

**Supplementary table 6: Outcome of other secondary endpoints in patients with TAE**

|  | High dose  n = 60 | Standard dose  n = 115 | p value | p* value |
| --- | --- | --- | --- | --- |
| Red blood cell transfusion units within 30 days(mean (SD)) | 5.8 (6.38) | 4.37 (5.00) | 0.309 | 0.243 |
| Length of hospital stay (mean (SD)) | 22.55(17,10) | 19.49(17.36) | 0.176 | 0.186 |
| Surgery operation within 30 days (%) | 9 (15.0) | 21 (18.3) | 0.676 | 0.869 |
| Therapeutic endoscopy within 30 days (%) | 5 (8.3) | 22 (14.7) | 0.078 | 0.120 |
| Repeating TA within 30 days (%) | 5 (8.3) | 6 (5.2) | 0.514 | 0.522 |
| ICU admission (%) | 13 (21.7) | 21(18.3) | 0.688 | 0.914 |

p value was calculated before IPTW; p* value was calculated after IPTW.

SD, standard deviation; ICU, intensive care unit; TA, transcatheter angiography; TAE, transcatheter angiographic embolization.

**Supplementary table 7: Outcome of other secondary endpoints in patients with targeted TAE**

|  | High dose  n = 58 | Standard dose  n = 102 | p value | p* value |
| --- | --- | --- | --- | --- |
| Red blood cell transfusion units within 30 days(mean (SD)) | 5.85 (6.76) | 4.56 (4.83) | 0.798 | 0.167 |
| Length of hospital stay (mean (SD)) | 25.47(18.05) | 21.11 (20.00) | 0.034 | 0.015 |
| Surgery operation within 30 days (%) | 12(20.7) | 21(20.6) | 1.000 | 0.743 |
| Therapeutic endoscopy within 30 days (%) | 7 (12.7) | 22 (21.6) | 0.199 | 0.120 |
| Repeating TA within 30 days (%) | 4 (6.9) | 7 (6.9) | 1.000 | 0.901 |
| ICU admission (%) | 13 (22.1) | 21 (20.6) | 0.842 | 0.668 |

p value was calculated before IPTW; p* value was calculated after IPTW.

SD, standard deviation; ICU, intensive care unit; TA, transcatheter angiography; TAE, transcatheter angiographic embolization.

**Supplementary table 8: Outcome of 30-day all-cause mortality and rebleeding rate in propensity score matching cohort**

|  |  |  | **Univariate** | | | | | | **Multivariate** | | | | |
| --- | --- | --- | --- | --- | --- | --- | --- | --- | --- | --- | --- | --- | --- |
|  | **No. of patients** | **No. of events** | | **HR(95%CI)** | | **P value** | | **aHR(95%CI)** | | | **P value** | |  |
| **30-day all-cause mortality** |  |  |  | |  | |  | | |  | |  |  |
| High dose | 79 | 23 | 1.366 (0.730, 2.557) | | 0.329 | | 1.471 (0.746, 2.899) | | | 0.265 | |  |  |
| Standard dose | 79 | 17 | 1 | | reference | | 1 | | | reference | |  |  |
| **30-day rebleeding rate** |  |  |  | |  | |  | | |  | |  |  |
| High dose | 79 | 41 | 0.784 (0.507,1.211) | | 0.272 | | 0.756 (0.481,1.189) | | | 0.226 | |  |  |
| Standard dose | 79 | 41 | 1 | | reference | | 1 | | | reference | |  |  |

CI, confidence interval; HR, hazard ratio; aHR, adjusted hazard ratio.

**Supplementary table 9: Outcome of 30-day all-cause mortality and rebleeding rate in patients without tumors**

|  |  |  | **Univariate** | | | | | | **Multivariate** | | | | |
| --- | --- | --- | --- | --- | --- | --- | --- | --- | --- | --- | --- | --- | --- |
|  | **No. of patients** | **No. of events** | | **HR(95%CI)** | | **P value** | | **aHR(95%CI)** | | | **P value** | |  |
| **30-day all-cause mortality** |  |  |  | |  | |  | | |  | |  |  |
| High dose | 71 | 11 | 0.879 (0.404, 1.913) | | 0.744 | | 0.888 (0.352, 2.243) | | | 0.801 | |  |  |
| Standard dose | 110 | 15 | 1 | | reference | | 1 | | | reference | |  |  |
| **30-day rebleeding rate** |  |  |  | |  | |  | | |  | |  |  |
| High dose | 71 | 30 | 1.251 (0.798,1.961) | | 0.330 | | 1.377 (0.860, 2.204) | | | 0.183 | |  |  |
| Standard dose | 110 | 52 | 1 | | reference | | 1 | | | reference | |  |  |

CI, confidence interval; HR, hazard ratio; aHR, adjusted hazard ratio.
